# Transplant Trial Watch

**DOI:** 10.3389/ti.2024.12853

**Published:** 2024-03-13

**Authors:** John M. O’Callaghan, John Fallon

**Affiliations:** ^1^ University Hospitals Coventry and Warwickshire, Coventry, United Kingdom; ^2^ Centre for Evidence in Transplantation, Nuffield Department of Surgical Sciences, University of Oxford, Oxford, United Kingdom

**Keywords:** randomised controlled trial, kidney transplantation, liver transplantation, hypothermic oxygenated machine perfusion, immunosuppression

To keep the transplantation community informed about recently published level 1 evidence in organ transplantation ESOT and the Centre for Evidence in Transplantation have developed the Transplant Trial Watch. The Transplant Trial Watch is a monthly overview of 10 new randomised controlled trials (RCTs) and systematic reviews. This page of Transplant International offers commentaries on methodological issues and clinical implications on two articles of particular interest from the CET Transplant Trial Watch monthly selection. For all high quality evidence in solid organ transplantation, visit the Transplant Library: www.transplantlibrary.com.

Randomised Controlled Trial 1 Preventing Kidney Transplant Failure by Screening for Antibodies Against Human Leucocyte Antigens Followed by Optimised Immunosuppression: OuTSMART RCT.

*by Stringer, D., et al. Efficacy and Mechanism Evaluation 2023; 10(5)*.

## Aims

This study aimed to assess the cost-effectiveness of preventing kidney allograft failure by optimising immunosuppression in human leucocyte antigen Ab+ patients.

## Interventions

Participants were randomised to receive either blinded standard care (SC) or unblinded biomarker-led care (BLC).

## Participants

2037 kidney transplant recipients >1 year post-transplantation.

## Outcomes

The primary outcome was time to graft failure following 43 months follow-up.

## Follow-Up

Up to 64 months.

## CET Conclusion

*by John O’Callaghan*

This is an extensive paper from 13 centres in the UK where anti-HLA antibodies were monitored after renal transplantation and immune suppression adapted in response. The study could not be blinded, clearly, for the purposes of monitoring and reviewing patients in the intervention arm. The study was large, including 2035 patients, also this number was required for the trial to be adequately powered to detect HR = 0.49. Patients were excluded if they received an HLA-incompatible transplant requiring desensitization. The prevalence and incidence rates of HLA Antibody positive patients were less than expected when the trial was planned. The primary outcome was therefore changed from transplant failure rate over 3 years to time to graft failure. The presence of donor-specific antibodies was associated with a higher risk of graft failure. However, the study found no evidence that biomarker-led care, with optimised immunosuppression in HLA antibody positive patients, delayed renal transplant failure. There was a significant reduction in rejection in the study group with biomarker-led care, but this did not carry through to improved graft survival. The development of non-donor specific antibodies was not associated with graft failure. The health economic analysis included in the paper demonstrates the biomarker-led care to be cost-ineffective.

## Trial Registration

EudraCT—2012-004308-36; ISRCTN—46157828.

## Funding Source

Non-industry funded.

Randomised Controlled Trial 2 Portable Hypothermic Oxygenated Machine Perfusion for Organ Preservation in Liver Transplantation (PILOTTM): A Randomized, Open-Label, Clinical Trial.

*by Panayotova, G. G., et al. Hepatology 2023 [record in progress].*

## Aims

To assess if HMP-O2 improves liver transplant outcomes compare to cold storage.

## Interventions

Livers were randomised to intervention, which was HMP-O2 on the Lifeport Liver Transporter device, perfused with Vasosol, or control, which was static cold storage.

## Participants

179 adult whole liver transplant recipients.

## Outcomes

The primary outcome was early allograft dysfunction (EAD) as defined by the Olthoff criteria. Secondary outcome measures were PNF, AKI, graft survival, biliary complications. Vascular complications and death. Additional exploratory outcomes were hospital LOS, ICU LOS, lactate clearance, bleeding, incisional hernia and SAEs.

## Follow-Up

12 months.

## CET Conclusion

*by John Fallon*

This large open labelled multi-centre randomised control trial is an exciting development in the field of liver HMP. The key strength of this work is that 43% (*n* = 27) of the HMP-O2 livers had continuous perfusion, having been placed on device at the donor. This is the first trial in liver HMP to do this and is an important development. Made possible by Organ Recovery Systems portable Lifeport Liver device, especially considering 81% travelled by air, a current limitation of the portable NMP devices. They demonstrated a nonsignificant reduction in EAD with 11% in HMP-O2 and 16% in SCS, while the finding is not significant it is in keeping with the 5 other published RCTs on HMP liver. The lack of significance may derive from the fact that within the intervention group only 24% were ECDs (including 5 DCD), upon sub-group analysis of these ECDs they find the reduction of EAD to be significant (20% in HMP-O2 and 33.3% in SCS *p* = 0.004). This is in keeping with previous large RCTs that the beneficial effects of HMP-O2 are amplified in the ECD cohort, especially in DCDs seen in Rijn et al’s 2021 trial published in the New England Journal who perfused only DCD livers. None of their secondary outcomes reach significance, but with PNF only occurring in the SCS group with 3 patients and a further 2 (*n* = 5 6.8%) went on to require re-transplant also due to ischaemic cholangiopathy. In HMP-O2 only 1 required retransplant, this was due to HAT. Biliary complications were nearly double in the SCS group (26.4% vs. 12.7%) which is impressive, but again this failed to reach significance. The trends are encouraging, but the lack of significance is disappointing, the trial having not been powered for overall EAD rates. An increase cohort size and a focus on EADs could have led to more dramatic results with potentially significance in many of the outcomes. An interesting note is the preservation fluid used in HMP-O2 was Vasosol, a UW-like solution with the addition of nitric oxide donors and vasodilators, this is the first HMP RCT across all organs to utilise this solution and could, in part be responsible for some of the beneficial trends. Unfortunately, the study was not sufficiently powered to compare continuous HMP-O2 with end-ischaemic HMP-O2 and SCS, the overall storage duration being comparable, but the percentage of that time being perfusion obviously being highest in the continuous group. They demonstrate safety and non-inferior efficacy of a novel portable device, which as it becomes more popular and people become more familiar with placing livers on device at retrieval more data should emerge on continuous HMP-O2, this trial was an important step.

## Jadad Score

3.

## Data Analysis

Per protocol analysis.

## Allocation Concealment

Yes.

## Trial Registration

Clinicaltrials.gov—NCT03484455.

## Funding Source

Industry funded.

## Clinical Impact Summary

*by John O’Callaghan*

This is a very interesting randomised controlled trial in liver transplantation, and an important step in the clinical implementation of a new device (the Lifeport Liver Transporter from Organ Recovery Systems). Hypothermic machine perfusion (HMP) with oxygenation was compared to standard static cold storage prior to transplant. The study was set up as a non-inferiority trial, and hence was smaller than it may have been if designed to demonstrate superiority of one treatment. The non-inferiority design was done specifically to obtain 510 (k) device clearance in the United States. Randomisation was stratified for MELD score and DCD status to maintain a distribution between study arms. Primary outcome was Early Allograft Dysfunction (EAD).

Approximately 40% of grafts in the HMP arm were put on the pump immediately at retrieval, demonstrating the portability of the device and safety in travel. Statistical analysis of the primary outcome proved non-inferiority of oxygenated HMP, but did not demonstrate superiority either. However, the rate of EAD in the control arm was far better than was expected; in the trial it was only 16%, when 30% had been used for the power calculation. When conducting a subgroup analysis of Extended Criteria Donor (ECD) livers, there was a significant benefit of oxygenated HMP, given the higher baseline risk of 33% EAD with static cold storage in this subgroup.

This trial report gives very reassuring information regarding the implementation of oxygenated HMP using this device, its ease of use, portability and safety. The benefit is seen in the ECD livers, and there is the possibility of benefits for standard criteria livers as well (for example PNF and biliary strictures) that may have been statistically significant and more clearly demonstrated in a larger trial.

